# Manipulation of Stacking Order in *Td*-WTe_2_ by Ultrafast Optical Excitation

**DOI:** 10.1021/acsnano.1c01301

**Published:** 2021-04-29

**Authors:** Shaozheng Ji, Oscar Grånäs, Jonas Weissenrieder

**Affiliations:** † Materials and Nano Physics, School of Engineering Sciences, KTH Royal Institute of Technology, SE-100 44 Stockholm, Sweden; ‡ Division for Materials Theory, Department of Physics and Astronomy, Uppsala University, SE-751 20 Uppsala, Sweden

**Keywords:** *Td*-WTe_2_, stacking order, photoinduced metastable phase, shear phonon, ultrafast electron diffraction

## Abstract

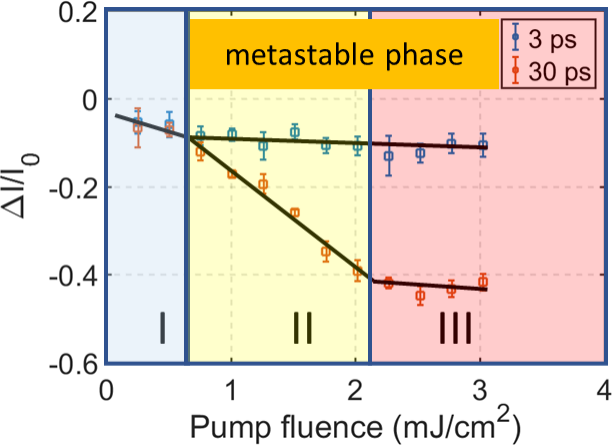

Subtle changes in stacking order of layered transition metal dichalcogenides may have profound influence on the electronic and optical properties. The intriguing electronic properties of *Td*-WTe_2_ can be traced to the break of inversion symmetry resulting from the ground-state stacking sequence. Strategies for perturbation of the stacking order are actively pursued for intentional tuning of material properties, where optical excitation is of specific interest since it holds the potential for integration of ultrafast switches in future device designs. Here we investigate the structural response in *Td*-WTe_2_ following ultrafast photoexcitation by time-resolved electron diffraction. A 0.23 THz shear phonon, involving layer displacement along the *b* axis, was excited by a 515 nm laser pulse. Pump fluences in excess of a threshold of ∼1 mJ/cm^2^ result in formation, with an ∼5 ps time constant, of a new stacking order by layer displacement along the *b* axis in the direction toward the centrosymmetric 1T* phase. The shear displacement of the layers increases with pump fluence until saturation at ∼8 pm. We demonstrate that the excitation of the shear phonon and the stabilization of the metastable phase are decoupled when using an optical pump as evidenced by observation of a transition also in samples with a pinned shear phonon. The results are compared to dynamic first-principles simulations and the transition is interpreted in terms of a mechanism where transient local disorder is prominent before settling at the atomic positions of the metastable phase. This interpretation is corroborated by results from diffuse scattering. The correlation between excitation of intralayer vibrations and interlayer interaction demonstrates the importance of including both short- and long-range interactions in an accurate description of how optical fields can be employed to manipulate the stacking order in 2-dimensional transition metal dichalcogenides.

The layered transition metal dichalcogenide *Td*-WTe_2_ has recently garnered significant scientific interest because of its intriguing physical properties, including large unsaturated magnetoresistance,^1^ Weyl semimetallicity,^2^ metallic ferroelectricity,^3,4^ and a nonlinear Hall effect.^5−7^ The electronic properties are closely tied to the crystal structure, especially the anisotropic atomic configuration within the layer and a stacking sequence that results in a break of centrosymmetry. Subtle manipulation of the stacking sequence allows for deliberate tuning of the electronic properties and may have relevance for future device applications.

*Td*-MoTe_2_, isostructural to *Td*-WTe_2_, exhibits a first order phase transition from an orthorhombic *Td* phase to a monoclinic 1*T*′ phase at temperatures in excess of 250 K.^8^ The *Td*-1*T*′ phase transition does not involve an intralayer structure change but is instead accomplished through an interlayer sliding mechanism.^8^ Such change in stacking order results in a recovery of centrosymmetry in the 1*T*′ phase. The electronic properties are correlated with the crystal structure, and the transition from *Td* to 1*T*′ phase is concomitant with a topological electronic transition from a Weyl semimetal to a topological trivial metallic state.^9^ The structural transition temperature of Mo_1–x_W_*x*_Te_2_ has been found to increase linearly with increasing W doping.^10,11^ For a long time, it was believed that *Td*-WTe_2_ did not exhibit a temperature driven *Td*-1*T*′ phase transition.^10,12^ However, most recently, such a phase transition was observed, by synchrotron X-ray diffraction, to occur at ambient pressure condition at 613 K.^11^ Another study using neutron scattering reports the transition at 565 K.^13^

Besides the temperature driven *Td*-1*T*′ phase transition, the symmetry of WTe_2_ can also be manipulated by means of electric fields,^3,14^ ultrafast laser pulses,^15^ and static pressure.^16,17^ Reference (15) reported that a metastable centrosymmetric and topologically trivial metastable phase can be reached within 20 ps after excitation by an ultrafast THz pulse. The formation of the metastable phase is considered to be intrinsically linked to the excitation of a 0.24 THz interlayer shear phonon propagating along the out-of-plane direction at the speed of sound that after traversing the thin film sample renders WTe_2_ in the metastable phase. The temporal evolution of the inversion symmetry was characterized by time-resolved second harmonic generation (SHG) spectroscopy.^15^ Excitation of *Td*-WTe_2_ by a 2.1 μm laser pulse at 10 MV/cm field strength was found to result in a suppression of the SHG within less than 2 ps. Similarly, it has been reported that the SHG response in the *Td*-MoTe_2_ phase can be suppressed at sub-picosecond time scales by femtosecond laser pulses at 800 nm and 2.6 μm.^18^ The suppression of SHG was interpreted as an ultrafast symmetry change induced by the ultrashort laser pulse. The time scale for completion of the structural transition, as shown in ref (15) is however significantly longer than that for the suppression of the SHG signal.^18^

Excitation of a coherent interlayer shear phonon at 0.24 THz frequency has been observed by intensive THz pumping of *Td*-WTe_2_, and the same study showed how the SHG intensity is modulated by the phase of the shear phonon, indicating a concomitant change in degree of centrosymmetry by the relative sliding of the layers.^15^ The shear phonon, an A_1_ optical phonon, can also be excited by a 800 nm pump laser pulse and has been studied using time-resolved reflection.^19^ Mode-resolved analysis from time- and angle-resolved photoemission spectroscopy (tr-ARPES) using 827 nm and 35 fs excitation pulses has revealed the excitation of no less than 5 distinct A_1_ optical phonon modes (at 0.24 THz, 2.41 THz, 3.57 THz, 4.00 THz, and 6.35 THz) at excitation fluence below what is needed to drive a phase change to the metastable phase.^20^ Generation of coherent phonons in absorbing semimetals may be explained through mechanisms including displacive excitation of coherent phonons (DECP)^21^ or by resonant impulsive stimulated Raman scattering (R-ISRS).^22,23^ Nakamura *et al*.^24^ proposed a model showing that both mechanisms depend on the pulse length and DECP is the dominating mechanism at the short-pulse limit. Tentatively the dominating mechanism for generation of coherent phonons in *Td*-WTe_2_ may depend on the pump wavelength, and therefore a change in excitation wavelength can result in different phonon dynamics. The observation of several coherent optical phonons in tr-ARPES, under IR pulse excitation with strong interband absorption, indicates a complicated scattering process between several phonon modes in the phonon thermalization process. The possible interplay between phononic excitation, thermalization, and lattice disorder with the phase transition need to be examined. As in the archetypal photoinduced phase transition in vanadium dioxide (VO_2_), it was recently presented that atomic disordering in the photoexcited state is central to the transition mechanism.^25^

Here we use time-resolved electron diffraction to determine the structural response of *Td*-WTe_2_ following excitation by an ultrafast laser pulse at 515 nm wavelength. The photoexcitation results in the activation of a layer shear mode (an ^1^A_1_ optical phonon) acting along the *b* axis and a transition to a metastable phase at a critical pump fluence. The transition to the metastable phase involves rearrangements in the stacking order driven by the ultrashort laser pulse. Through a combination of experimental diffraction results and time-dependent simulations in the framework of density-functional theory (DFT), we will discuss the correlation of the excited phonons and the formation of the metastable phase in *Td*-WTe_2_. Through studies of samples with defects that show a suppressed shear phonon, the excitation of the shear phonon can be decoupled from the formation of the metastable phase. Results from diffuse scattering and dynamic DFT simulations allow us to propose a mechanism for the transition where transient local disorder is prominent before settling at the atomic positions relevant for the metastable phase.

## Results and Discussion

### Crystal Structure of *Td*-WTe_2_

**Figure 1:**
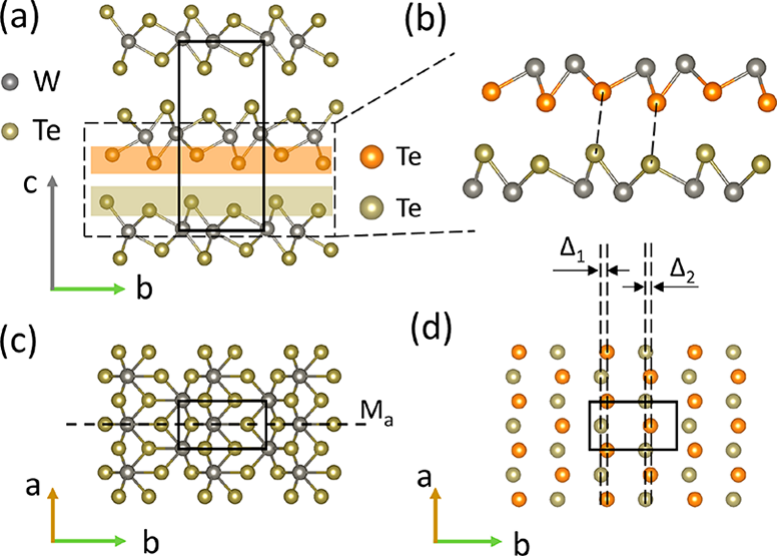
Illustrations of the *Td*-WTe_2_ crystal structure. The unit cell is indicated by solid rectangles. (a and c) Projections along the *a* and *c* axes. (b) Enlarged view of the dashed rectangle indicated in part a. The Te atoms in part b are color-coded according to their positions in the *Td*-WTe_2_ unit cell, with lower Te atoms in the middle layer in orange and upper Te atoms in the lower layer in green. (d) Projection along the *c* axis including only the color-coded Te atoms in part b. The dashed lines indicate the *b* axis displacements (Δ_1_ and Δ_2_) between the Te atoms in the adjacent layers.

We begin by orienting ourselves in the ground-state crystal structure of the orthorhombic *Td*-WTe_2_ phase. Figure 1 shows projections of the atomic structure with some relevant features indicated. The solid rectangles in Figure 1a, c, and d indicate the unit cell in the out-of-plane and the in-plane directions. The dashed line in Figure 1c shows the mirror symmetry plane (M_a_). Distortions of the [WTe_6_] octahedra result in an anisotropic in-plane atomic configuration and leave the atomic layers of Te and W buckled in the out-of-plane, *c*, direction (Figure 1b). This results in the formation of ridges and troughs in the WTe_2_ layers. The layer stacking sequence is governed by an organization of these ridges and troughs with lattice displacements Δ_1_ and Δ_2_ in the *b* direction for the Te atoms compared to the adjacent layers in the unit cell.^26^ The dashed lines in Figure 1d show the Δ_1_ and Δ_2_ displacements along the *b* axis in a projection along the *c* axis. Similar stacking shifts exist between the top and middle WTe_2_ layers of the unit cell, but in the opposite direction. The stacking arrangement in the out-plane direction of the *Td*-WTe_2_ phase results in a break of inversion symmetry and formation of a net electric polarization in the out-plane direction.^27^ A nonpolar centrosymmetric transition state (1T*) can be reached by translating the middle layer of the unit cell by (Δ_1_ + Δ_2_)/2 toward the −*b* direction.^27^ This corresponds to a shift of the layers of 0.37 Å.^28^

### Excitation of Shear Phonon

**Figure 2:**
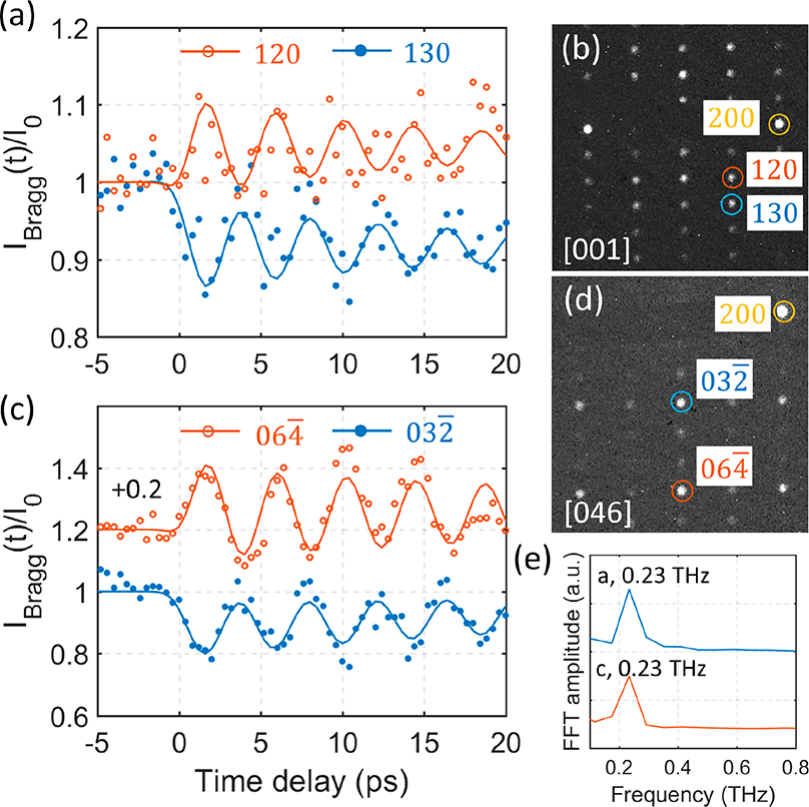
Excitation using 515 nm at 1.9 mJ/cm^2^ pump fluence on a 0.44 IMFP (∼41 nm) sample, below the threshold for initiating a phase transition. Intensity evolution of selected diffraction spots at the [001] (a) and the [046] (c) zone axes. (b and d) Experimental diffraction patterns at the [001] and [046] zone axes, respectively. (e) Fourier transforms of the 130 and the 032̅ intensity traces in parts a and c.

The distorted crystallographic structure will arguably influence the structural response of the material upon photoexcitation. We performed time-resolved selected area electron diffraction on a WTe_2_ thin film sample at a different zone axis. The structural response can be traced by analyzing the intensity evolution of diffraction spots in the diffraction pattern indicated in Figures 2b and d. As reported in the aforementioned tr-ARPES study, electronic excitation by an ultrashort IR laser pulse may result in coherent excitation of several A_1_ optical phonon modes.^20^ Not surprisingly we observe intensity oscillation of selected diffraction spots in the time-resolved electron diffraction results (Figure 2). The intensities of the spots oscillate at a frequency of 0.23 THz (Figure 2e) when excited at a fluence of 1.9 mJ/cm^2^. The sample thickness is here 0.44 inelastic mean free path (IMFP), or ∼41 nm, as characterized from electron energy loss spectroscopy (EELS). The frequency fits the ^1^A_1_ optical phonon frequency observed by Raman spectroscopy.^29^ By lowering the temperature to 110 K, the frequency increases to 0.27 THz (Supporting Information Figure S1). The temperature dependence of the phonon frequency can be described by applying a model of anharmonic optical phonon decay into acoustic phonons with the same frequencies and opposite momenta.^19,30,31^ The frequencies observed here at 110 K and at room temperature are consistent with the time-resolved reflection results reported in ref (19). The pump laser pulse width used in the experiment is 300 fs which implies that optical phonons with a higher frequency than ∼3 THz will not be coherently excited.^24^ From time resolution limitations, we did not observe excitations of other optical phonons in the results. A combination of an exponential decay with a sub-picosecond time constant and the 0.23 THz phonon are used to fit the temporal trace of the diffraction intensity in Figure 2 (see Supporting Information for details on the fitting procedure).

The excited ^1^A_1_ optical phonon, which is called the “shear” phonon in ref (15), can be described as an antiphase motion of adjacent layers of WTe_2_ along the *b* axis.^15^ The initial shear direction of the layers is illustrated in Figure S2b. To conclusively determine that the observed phonon is indeed a shear phonon of this symmetry, we analyzed diffraction patterns at both the [001] and the [046] zone axes (Figure 2). At the [001] zone axis, the diffraction spots 120 and 130 show an out-of-phase intensity oscillation with a frequency of 0.23 THz as shown in Figure 2a. In Figure 2c, the same out-of-phase oscillation is observed for the 032̅ and the 064̅ diffraction spots collected at the [046] zone axis. Calculated structure factors shown in Supporting Information Figure S2, modulated by displacements along the *b* axis inflicted by the shear phonon, are consistent in phase with the experimental results for the different diffraction spots shown in Figure 2 and Figure S1. In the structure factor calculations, we initially move the middle layer in the unit cell in Figure 1a toward the negative *b* direction followed by a motion toward the positive *b* direction. The middle layer then oscillates around a new quasi-equilibrium position at a negative *b* displacement. In accordance with this structure model, the average intensity over a period of 120 and 064̅ increases while 130 and 032̅ decrease in intensity. The oscillation in intensity of the diffraction spots can be described by a cosine function which is consistent with a mechanism described by the DECP model where the photoexcitation of the electronic system results in formation of new quasi-equilibrium positions of the lattice.^21^ The shift in quasi-equilibrium lattice position gives rise to a coherent vibration of A_1_ symmetry. The new quasi-equilibrium stacking positions are established within one period of the shear phonon, in agreement with the DECP model. This is what is observed in Figure 2 where the adjacent layers shear around the quasi-equilibrium position like a damped vibration with the first period exhibiting the largest amplitude. Further, below 0.5 mJ/cm^2^ we observe a linear dependence of the shear phonon amplitude as a function of pump fluence in a WTe_2_ sample of thickness <10 nm (Supporting Information Figure S4). A similar near linear increase in shear phonon amplitude as a function of pump fluence has been reported in *Td*-MoTe_2_.^18^ A linear dependence of the oscillation amplitude with excitation fluence is also in agreement with the DECP mechanism.^21^ In contrast to our results, ref (20) reports a sinusoidal response to the shear phonon in the band-structure modulation. The low excitation fluence in the study (0.1 mJ/cm^2^) will result in diminishing lattice displacement that tentatively can render discrimination between sine and cosine modes of oscillation difficult in tr-ARPES.

### Formation of a Metastable Phase

**Figure 3:**
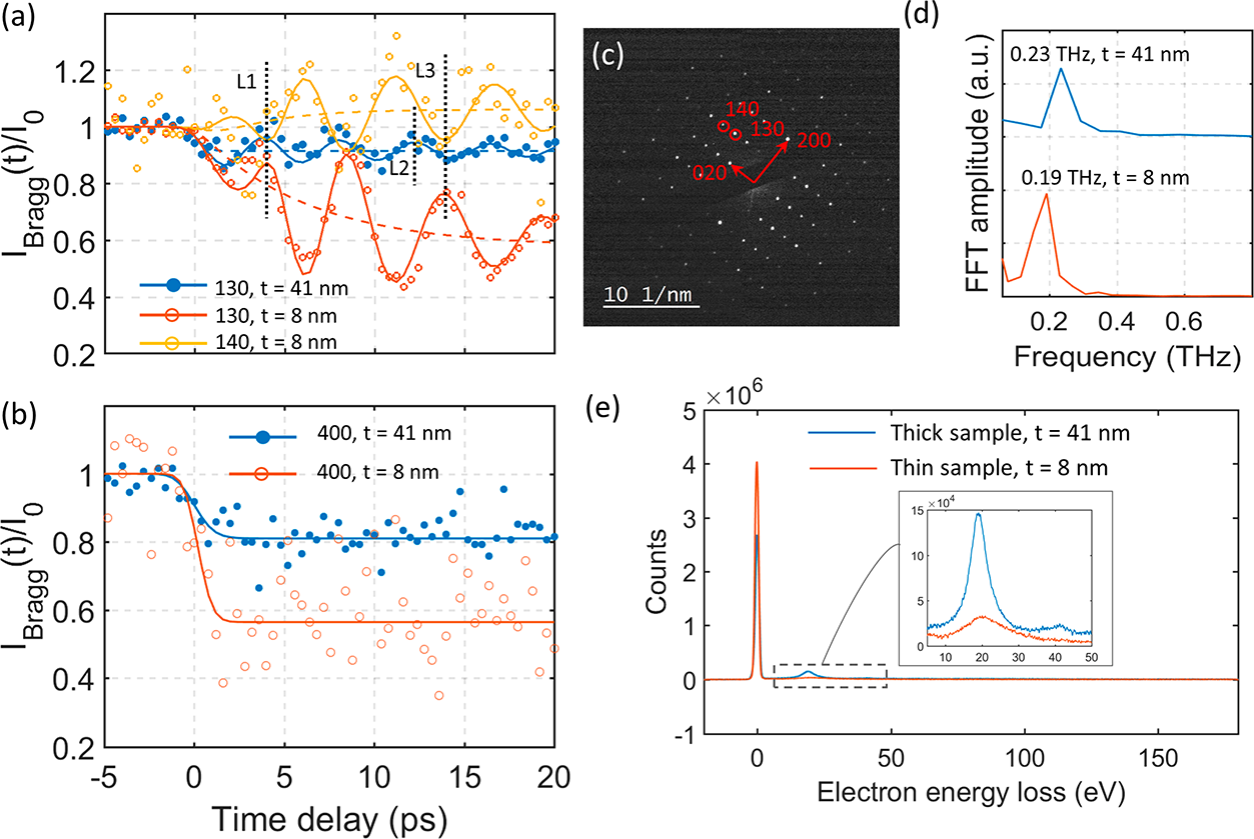
(a and b) Evolution in temporal diffraction intensity of several diffraction spots from a thick (41 nm) and a thin sample (8 nm) at 1.9 mJ/cm^2^ pump fluence and 515 nm wavelength. The vertical dashed lines L1–L3 in part a are guides to the eye in comparing oscillation periods after an ∼4 ps delay. (c) Diffraction pattern at the [001] zone axis. (d) FFT amplitudes from data in part a. The thin (thick) sample shows oscillation with a frequency of 0.19 THz (0.23 THz). (e) Electron energy loss spectroscopy from the two samples with the corresponding estimated sample thicknesses (41 and 8 nm).

A different structural response is observed when using the same 1.9 mJ/cm^2^ laser fluence on a thinner sample (thickness of 0.09 IMFP, ∼8 nm, characterized from EELS as shown in Figure 3e). These results are summarized in Figure 3. As a comparison we have included the 130 spot intensity trace of the 41 nm sample previously shown in Figure 2a. A 0.19 THz frequency was obtained from the Fourier analysis of the 130 and 140 diffraction intensity oscillations obtained from the 8 nm sample, as shown in Figure 3d. This is a slightly lower frequency than the observed 0.23 THz for the 41 nm thick sample. From the 130 and 140 traces it is also clear that another, slower, process is active in the thin sample, in addition to the shear oscillation. A cosine function and two exponential functions must be used to fit the intensity traces of the 130 and 140 spots. The exponential models a fast exponential decay with a sub-picosecond time constant and a slower decay/rise time with ∼5 ps time constant (see Supporting Information for details). The solid lines in Figure 3a are results from the fitting procedure. The yellow and red dashed lines in Figure 3a indicate the slower intensity changes of the 130 and the 140 spots. The 130 and the 140 spots show an opposite change in intensity for the slower process. At 20 ps delay, the 130 spot intensity has decreased approximately 40% while the 140 spot intensity has increased approximately 10%. The changes in diffraction intensities can be rationalized from the formation of a metastable phase with a modified crystal structure. These results cannot be explained by lattice heating since the intensity of some diffraction spots increased, *e.g*. the 140 spot shown in Figure 3a. If this was an effect purely due to lattice heating, we would expect that all the diffraction spots should decrease in intensity (due to the Debye–Waller effect).

The in-plane anisotropic atomic configuration illustrated in Figure 1c is reflected in different detected dynamics of the *a* and *b* axes. The intensity of the 400 spot in both the thin and the thick samples shows only a sub-picosecond exponential decay (Figure 3b) which is interpreted as a result of rapid electron–phonon coupling. The solid lines in Figure 3b are the fitting results considering the sub-picosecond exponential decay and the instrument response time. At a laser fluence of 1.9 mJ/cm^2^, the intensity of the 400 spot in the thick sample decreases 20% while in the thin sample the decrease is approximately 40%. It is not surprising that the thin sample is more strongly excited since the optical penetration depth is around 20 at 515 nm.^32,33^ The thin sample will on average absorb more energy per unit volume compared to the thick sample. No intensity oscillation can be detected for the 400 diffraction spot. This supports the interpretation that the shear phonon is associated with a displacement along the *b* axis. Furthermore, the ∼5 ps time constant for the change in intensity observed at the 130 and 140 spots in the 8 nm sample was not detected in the 400 spot. Based on this result, it can be inferred that the process with the ∼5 ps time constant is a result of a structural change along the *b* axis. The changes in intensity for the 130 and the 140 spots associated with the ∼5 ps time constant process are similar in sign to the changes in intensity induced by the shear phonon. The 130 spot decreases in intensity, and the 140 spot increases in intensity. This is also true for the 120 and the 150 spots. Taken together with the results in refs (15) and (34), this leads us to conclude that the process involves rearrangements in the layer stacking along the *b* axis that can be modeled such that the middle layer of the unit cell shears toward the negative *b* direction to reach a new equilibrium stacking position with a time constant of ∼5 ps. The phase transition to the metastable phase is completed approximately 20 ps after photoexcitation and persists over 200 ps (Figure S3). A transition to the metastable phase is not evident for the 41 nm thick sample at 1.9 mJ/cm^2^ pump fluence (Figure 3a). The thick sample is on average less intensively pumped, and it appears that a critical pump fluence may be required for inducing the transition to the metastable phase.

### Decoupling of the Shear Phonon and the Phase Transition

A careful comparison of the intensity trace from the 130 spot of the 41 nm thick sample shows that the first minima of the shear phonon induced oscillation in intensity coincides with the minimum intensity of the trace. This is observed for both zone axes in Figure 2. In the thin (8 nm) sample the picture is different; the first minima of the intensity oscillation of the 130 spot exhibits an approximately 20% decrease in intensity while the decrease in intensity for the second minima is 50%. The shear phonon amplitude decays after two intensity oscillation periods (around 8 ps). During the first period of the oscillation, the shear frequency is almost the same in the thin and thick samples. However, a softening of the shear phonon in the thin sample becomes apparent during and after the formation of the metastable phase. The vertical dashed lines L1–L3 in Figure 3a serve as guides to the eye in comparing the oscillation periods after the first period. This result indicates that the photoexcited shear phonon in the thin sample under excitation conditions driving WTe_2_ through a phase transition is not acting as a trivial harmonic oscillator but also reflects structural aspects. The transition to the metastable phase appears to influence both the shear phonon amplitude and its frequency. At 20 ps delay when the lattice has reached quasi-equilibrium positions, the intensity of the 130 spot is at around 0.6I_0_. The shear displacement at the first minima (at around 2 ps delay) that is associated with the shear phonon is smaller than the displacement relevant for the metastable phase completed at 20 ps which indicates that the formation of the metastable phase cannot be explained by a displacive excitation mechanism.

**Figure 4:**
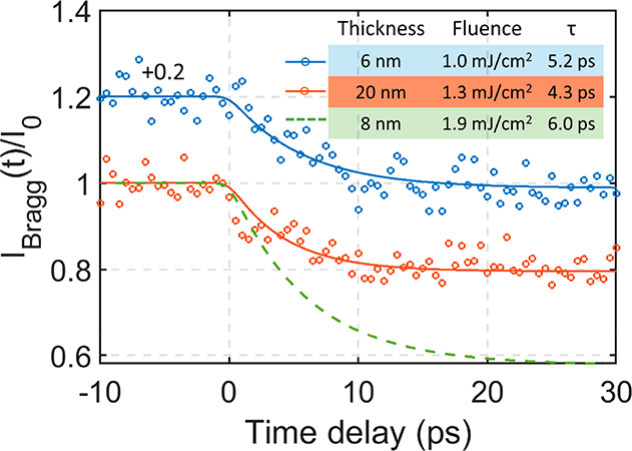
Intensity evolution of the 130 diffraction spot following photoexcitation by 515 nm for samples with suppressed shear phonon dynamics and different thicknesses. The thickness was estimated by EELS spectroscopy. The pump fluence and time constants obtained from the fitting procedure are listed in the inserted table. The green dashed line shows the corresponding decrease in 130 intensity extracted from the fit in Figure 3a.

Further evidence for a decoupling of the shear phonon and the phase transition can be found in results obtained from samples where the shear phonon is suppressed. TEM observations show that nonuniform samples that host defects often do not exhibit a strong shear phonon response in the temporal traces (TEM image in Supporting Information Figure S5). Shear displacements are known to be strongly damped by defects and boundaries.^35^ The intensity evolution of the 130 spot from such defective samples is shown in Figure 4. Two sample thicknesses are included, 0.06 IMFP (∼6 nm) and 0.21 IMFP (∼20 nm). The shear phonon induced intensity oscillations are weak in both samples, but the slow exponential decay associated with formation of the metastable phase is clearly observed. The slow exponential decay of the 130 spot extracted from the fit in Figure 3a from the 8 nm sample is included as a comparison (green dashed line). The time constants of the exponential decay from the fitting procedure are listed in the table inserted into Figure 4. All time constants are within the range of 4–6 ps. The combination of these results infers that the formation of the metastable phase by 515 nm excitation is not a result of transportation of a shear phonon wave along the *c* axis, as was suggested from a single point matching of sample thickness, sound velocity, and time for completion of the phase transition in the THz driven phase transition in ref (15). For a sample thickness below 20 nm, the sample is almost uniformly excited by the laser pulse throughout the out-of-layer dimension since the penetration depth is around 20 at 515 nm excitation wavelength.^32,33^

### Pump Fluence Threshold of the Phase Transition

**Figure 5:**
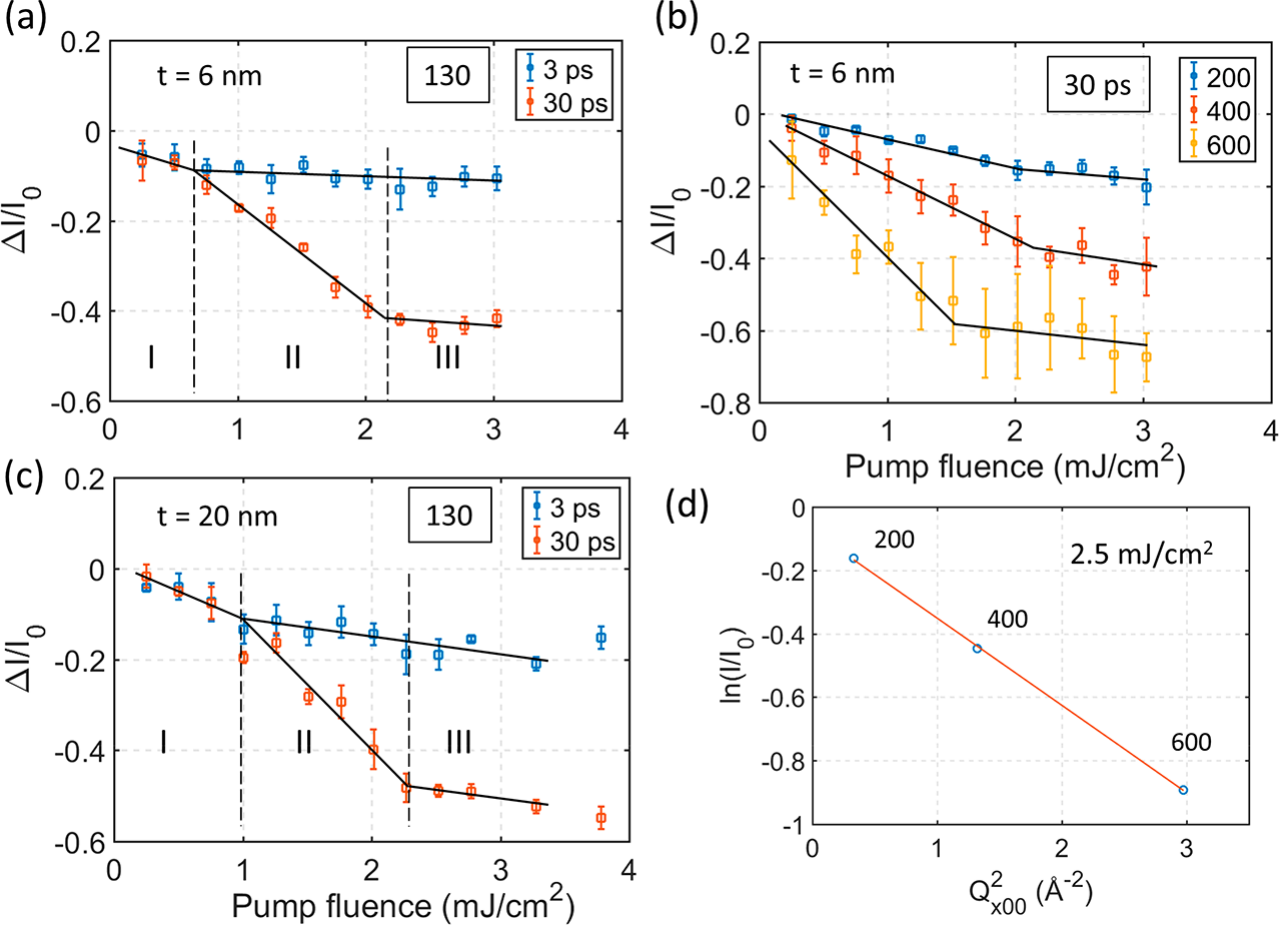
Pump fluence-dependent diffraction intensity change. (a and c) 130 diffraction intensity at 3 and 30 ps delay from samples of thicknesses 6 and 20 nm. (b) a00 diffraction intensity at 30 ps from a 6 nm thickness sample. The solid lines in parts a–c are drawn as a guide to the eye. (d) Diffraction intensity of a00 as a function of Q^2^ at 2.5 mJ/cm^2^ pump fluence. The red line is the result from a linear fit.

The results in Figure 3a indicate that there may be a threshold pump fluence necessary to induce the formation of the metastable phase. In a pump fluence dependence analysis, we studied two samples with suppressed shear phonon dynamics with thicknesses of 0.06 IMFP (∼6 nm) and 0.21 IMFP (∼20 nm), the same samples as in Figure 4. The strong suppression of the shear phonon allowed us to ignore its influence on the intensity evolution and thereby reduce the uncertainty of the fitting procedure. Photoexcitation at these sample thicknesses is, as mentioned above, nearly uniform in the out-of-plane direction. By comparing the intensity of the 130 spot at 3 and 30 ps delay, we can estimate the decay associated with the formation of the metastable phase under the assumption that the electronic carriers have reached a Fermi–Dirac distribution and that the temperatures of the electronic system and the lattice system are similar.^20^ The results from this study are summarized in Figure 5. Figures 5a and c show that the fluence-dependent intensity decay trace of spot 130 can be divided into three regions (I, II, and III) in both samples. In region I, the diffraction intensity at 3 and 30 ps is nearly the same and both decrease similarly with increasing laser fluence. In region II, a gap opens between the intensity at 3 and 30 ps. This gap increases linearly with laser fluence. The transition between region I and II for the sample in Figure 5a is around 0.7 mJ/cm^2^ while for the sample in Figure 5c, the corresponding threshold fluence is around 1 mJ/cm^2^. This difference in threshold may be attributed to the different sample thickness. For pump fluences in excess of 2.3 mJ/cm^2^ a change in slope is observed again (Region III). Now the intensity at 30 ps delay decreased only slowly with fluence and with a similar slope as the 3 ps trace. This can also be observed from the fact that the gap between the 3 ps and the 30 ps traces becomes almost constant. From these results we can conclude that there is a fluence threshold for the phase transition to commence. This threshold is 0.7 mJ/cm^2^ in a 6 nm sample and 1.0 mJ/cm^2^ in a 20 nm sample. Above this threshold a phase transition that includes a shear displacement of adjacent layers occurs. The quasi-equilibrium shear displacement increases with pump fluence. At a fluence of approximately 2.3 mJ/cm^2^, the shear displacement of the metastable phase reaches saturation (Region III). The shear displacement of adjacent layers can be estimated from the intensity gap between the 3 ps and the 30 ps traces in Region III by considering the change in structure factor as a function of shear of the middle layer of the unit cell toward the negative *b* direction (Supporting Information Figure S6). The Debye–Waller effect is estimated from the intensity decay at 3 ps. In structure factor simulations we extract a saturation shear displacement of ∼8 pm by considering four different diffraction spots in the diffraction pattern from the 6 nm thick sample (Supporting Information Figure S6). A 8 pm shear displacement is similar to what was reported in ref (15), indicating that the metastable structure is similar for optical and THz excitation. The sample has however not quite reached the 37 pm displacement required for reaching the centrosymmetric 1T* phase.

As mentioned in context to Figure 3b, the intensity of the a00 diffraction spots will decrease with a sub-picosecond time constant without showing any signs of a second slower process. This is regardless if the sample undergoes a phase transition or not. Pump fluence-dependent intensity changes of selected a00 spots in a 6 nm thick sample at 30 ps are shown in Figure 5b. At fluences below approximately 2 mJ/cm^2^, we observe a linear decrease in the 200 and the 400 intensities with pump fluence. The relative decrease of the 400 spot is larger than that of the 200 spot. The slope of the intensity decrease is reduced at fluences above 2 mJ/cm^2^. A similar behavior is observed for the 600 spot, but the change in slope is observed already at a fluence around 1.5 mJ/cm^2^. This discrepancy may tentatively be explained by the larger error bars associated with the 600 spot. The observation of a saturation, similar to what was reported for the 130 spot, indicates that the structure is less responsive to an increase of pump fluence in both the *a* and *b* axes in excitation Region III. The relation between ln(I/I_0_) and Q^2^ for a00 at 2.5 mJ/cm^2^ pump fluence is shown in Figure 5d. ln(I/I_0_) is linear with Q^2^ which is consistent with what is expected from the Debye–Waller effect.^36^

### Mechanism for the Phase Transition

Now we can summarize the structural response to the photoexcitation of WTe_2_ by a 300 fs laser pulse at 515 nm. A shear phonon at around 0.23 THz, which involves the shear displacement of adjacent layers in the unit cell along the *b* axis, is excited. Other optical phonons may be excited incoherently and contribute to the observed sub-picosecond decay in diffraction intensity. A metastable phase is formed when the pump fluence exceeds a critical threshold. The transition to the metastable phase is associated with an ∼5 ps time constant and involves reorganization of the layer stacking sequence by a shear displacement for every other layer along the *b* axis. The layer displacement increases linearly with pump fluence until a saturation displacement is reached at around 8 pm. In samples with a pinned shear phonon, the metastable phase transition is still readily observed. The shear displacement at the first minimum (at around 2 ps delay) that is associated with the shear phonon is smaller than the displacement relevant for the metastable phase completed at 20 ps which indicates that the formation of the metastable phase cannot be explained by a displacive excitation mechanism.

**Figure 6:**
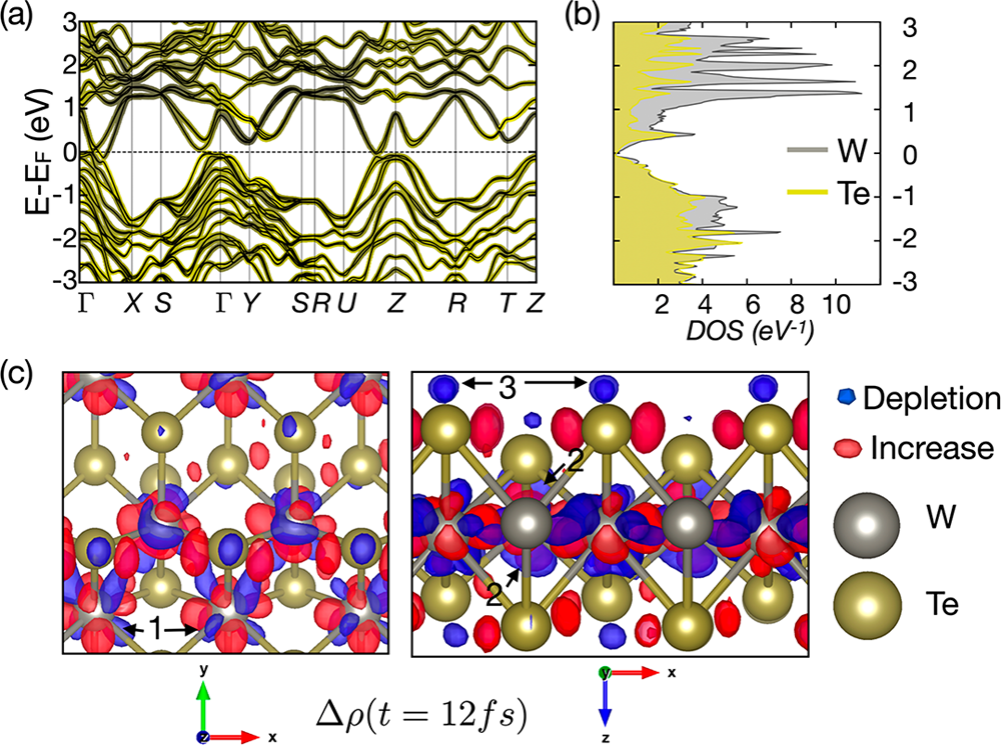
(a) Band-structure and density of states of the *Td-*WTe_2_ phase. The color gradient in the left panel indicates the character of the band according to a projection of the wave functions on to W (gray) and Te (yellow) sites. (b) Site projected density of states with the same color scheme. Excitation for a photon energy in the range of 2.4 eV excites carriers from bonding to antibonding states. (c) Electron redistribution in the *x*–*y* plane (left) and the *x*–*z* plane (right), extracted from the TD-DFT simulations where *t* = 12 fs. The iso-value for plotting the surfaces is 0.0015 electrons/Å^3^.

To further the understanding of the structural response following photoexcitation, we provide insights into the early stages of the absorption process from first-principles simulations (see the Computational Method section for details). We start by investigating the ground-state electronic-structure of the material using density-functional theory calculations. Figure 6a depicts the band-structure and density of states of *Td*-WTe_2_. An optical absorption process will be to first order excite electrons occupying states in the valence band to states in the conduction band. Our photon energy requires the energy difference between the valence and conduction-band states to be ∼2.4 eV. The electronic states involved in the photoexcitation can be deduced from the projection of the density of states onto atomic types (Figure 6b), as well as the color gradient in the band-structure plot, indicating atomic character (Figure 6a). Electrons are excited from hybridized W–Te bonding states, owing to the covalency of the material, to antibonding states, with some involvement of nonbonding localized states (bands with little energy-dispersion). The level of hybridization within the bands is indicated by the color gradient in Figure 6a. Electron–electron scattering will typically allow for electrons to move toward the conduction-band edge in the femtosecond time scale, and no electron–hole pairs are expected to be trapped for picoseconds due to the metallicity of the sample, although the possibility exists around, *e.g*., the Y-point in the Brillouin-zone.

**Figure 7:**
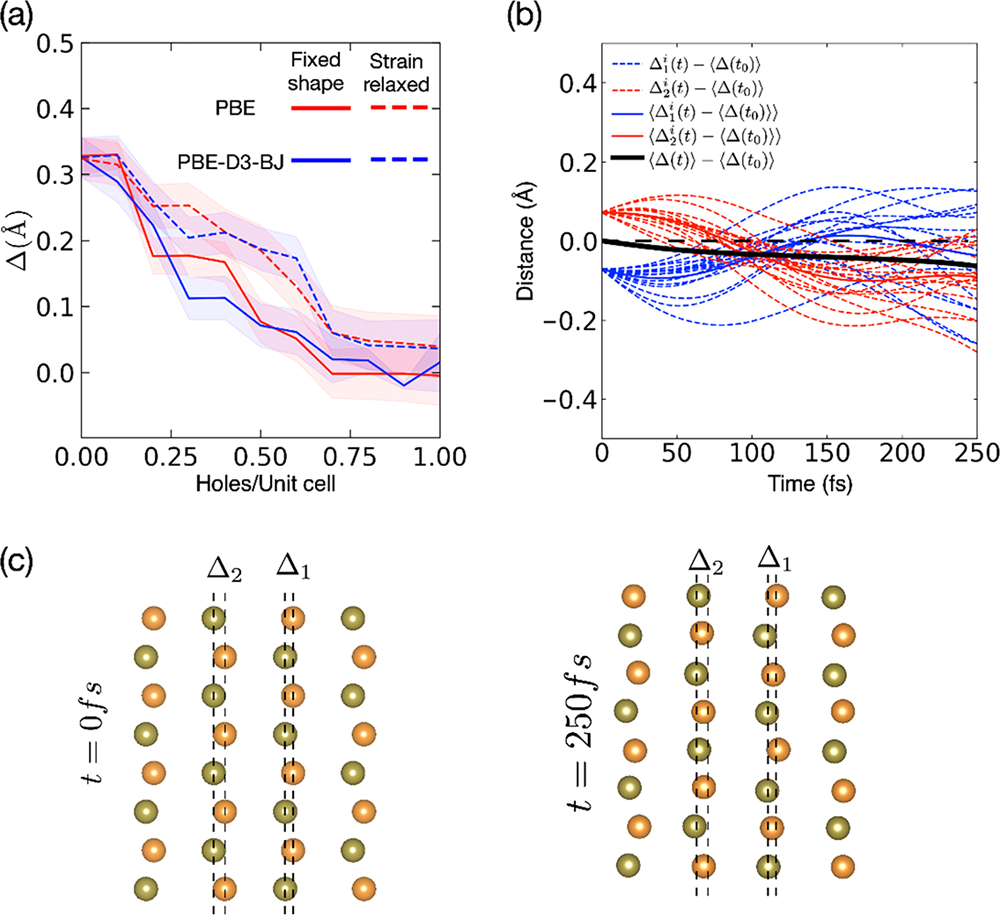
Part a indicates the Δ-value for the equilibrium geometry with different levels of hole-doping. We compare two different treatments of exchange and correlation within DFT (PBE and PBE-D3-BJ) and the effects of strain relaxations. Part b shows the change in Δ as a function of time; the optical drive induces a significant disorder in the lattice. Part c visualizes the individual change in position of the Te-atoms in adjacent layers in relation to Δ _1_ and Δ _2_ (see caption of Figure 1 for details).

Further, we proceed to investigate the out-of-equilibrium electron distribution using time-dependent density-functional theory in the real-time formulation. Figure 6c shows the density-differences at t = 12 fs: Arrows in the left panel indicated with 1 refer to density depletion along the W–Te bond. Arrows in the right panel indicated with 2 indicate that density is redistributed from within the W–Te sheets, to being more localized around the W atoms. Arrows indicated with 3 show the depletion of antibonding states in the vicinity of the Te-atom, between adjacent WTe_2_ layers. At 12 fs, the lattice response is still weak. And we can study the isolated effects on the electron density with little involvement of bond-oscillations. While we stress that our sample remains neutral during our optical pump, we see that the changes in electronic structure are similar to those observed upon hole-doping. The depletion of antibonding Te-states is known to stabilize the centrosymmetric 1T* phase.^34^ To assess if this scenario is possible in picosecond time scales, not allowing for significant volumetric change, we simulate the effect of hole-doping on the *b* axis displacement (Δ) with constant cell volume. We relax the ionic positions with fixed cell shape (not allowing for strain relaxation) and with strain relaxation. The result, displayed in Figure 7a, is consistent with previous calculations using full relaxations^34^ in that an effective hole-doping of the valence band modifies the equilibrium *b* axis displacement gradually toward the 1T* phase.

When the pump fluence is higher than a critical threshold, a metastable phase with a corresponding new quasi-equilibrium stacking position for the adjacent WTe_2_ layers along the *b* axis is formed with an ∼5 ps time constant. Theory and experiment show that hole-doping may stabilize a phase transition by a layer sliding mechanism.^12,14,34^ The ∼5 ps time constant of the transition process is independent of sample thickness which is in contrast with the reported mechanism for THz excitation where the phase transition was interpreted as completed once the shear wave has traversed the sample thickness.^15^ Further, formation of a metastable phase is observed also in samples where the shear phonon is heavily suppressed indicating that the formation of the metastable phase is not a direct result of excitation of a large amplitude shear phonon. An ultrafast transient reflectivity study of *Td*-WTe_2_ reports two recovery time constants following photoexcitation,^37^ a fast recovery process occurring at sub-picosecond time scales and a slow, several picosecond, recovery process. The fast process was reported as due to thermalization of the electronic subsystem through rapid electron–phonon coupling. This process is reflected in the sub-picosecond intensity decay observed in our diffraction data. The slow process which is reported at ∼5 ps at room temperature is interpreted as phonon-assisted electron–hole recombination between electron and hole pockets.^37^ The time constant for the slow process is close to the observed time constant for the formation of the metastable phase. However, the lifetime of photoexcited coherent optical phonons at 3.57 (^3^A_1_ – cage stretching mode) and 6.35 THz (^9^A_1_ – motion of W atoms in plane) observed by tr-ARPES is also similar to the time scale for the formation of the metastable phase.^20^ Based on these results, it can be inferred that phonon thermalization is involved in the formation of the metastable phase.

Using a combination of TD-DFT and Ehrenfest molecular dynamics, we track the lattice excitation for 250 fs. In our simulations, we employ a rectangular pulse shape, represented by continuous illumination between t = 0 and t = 250 fs; see the Supporting Information for details. Optical pulses act predominantly on the electronic system, where electron–phonon coupling facilitates energy transfer to vibrational modes; this can be seen in Figure 7, where the change in Δ’s is depicted. The first lattice response is an effect of the weakened bond between W and Te, resulting in a significant excitation of the W–Te bond-stretching mode. This can be seen as a breathing-like mode of the distorted Te octahedron surrounding the W atom. The phonon excitation, because of the electron–phonon coupling, will result in the sub-picosecond intensity decay observed in the electron diffraction results. Phonon–phonon scattering subsequently induces local disorder in the cage-structure. Recently it was argued that local disorder is central to ultrafast phase transitions, while they are hard to probe with diffraction due to the locality of the response.^25^ In our study this is reflected in the observed sub-picosecond intensity decrease in the diffraction spot as shown in Figure 3b and the increase in diffuse scattering as shown in Figure S7. Through our calculations we see that similar to what is reported for VO_2_,^25^ WTe_2_ exhibits prominent transient disorder on short time scales, comparable to phonons. In order to quantify the change in lattice asymmetry, we monitor the value of Δ (see Figure 1 for definition) as a function of time. The supercell used in the molecular dynamics simulation encompasses 16 independent values of Δ of each kind. The excitation of the W–Te bond-stretching mode further induces a rotational dynamic of the Te-cages, resulting in a decrease in Δ. In Figure 7b we display the value of Δ as well as the averages as a function of time. Figure 7c depicts the positions of the Te-atoms used to quantify Δ at t = 0 fs and t = 250 fs. On average the highly nonequilibrium vibrational state is more symmetric than the original phase. We hypothesize that the rapid quench in second-harmonic generation seen in, *e.g*. refs (15 and 18) is due to loss of order in relation to the polarity of the sample. Further, since the parent phase is stabilized by fitting the ridges of one layer to the troughs of the subsequent layer, the potential energy landscape is modulated. The photoinduced local disorder and change in local cage-structure will influence the quasi-equilibrium stacking order, and the path to the stacking order of the metastable phase may proceed through thermalization of the observed photoexcited disordered state rather than an immediate layer displacement driven by the shear phonon. Combined with an effective hole-doping of the valence band (see Figure 7a), as driven by the optical field, such modulation could facilitate the shear displacement to the metastable phase. By assuming 50% absorption in a 20 nm thick sample we can estimate the initial hole concentration to ∼0.2 per unit cell for 1 mJ/cm^2^ pump laser fluence. Such hole concentration is sufficient to drive a similar shear displacement to what is observed experimentally (Figure 7a).

## Conclusions

In summary, we provide a description of the structural response of *Td*-WTe_2_ following ultrafast laser excitation with interband absorption. The response includes shear phonon excitation and formation of a metastable phase, with an approximately 5 ps time constant. Both processes involve sliding of adjacent WTe_2_ layers along the *b* axis. We show that the shear phonon and the phase transition are decoupled processes. This conclusion comes from similar detected phase transition time constants for samples with different thicknesses and that the phase transition still takes place in samples with heavily suppressed shear phonon dynamics. Simulations observe a strong disorder in the [WTe_6_] octahedra on short time scales. This is corroborated by the sub-picosecond intensity decay observed in experimental electron diffraction and the rapid rise in diffuse scattering. Since *Td*-WTe_2_ is stabilized by alignment of the ridges in one layer with the troughs in adjacent layers, the disorder of the [WTe_6_] octahedra will result in a modulation of the potential energy landscape determining the relative coordination of the layers. The path to the stacking order of the metastable phase can proceed through thermalization of the observed photoexcited disordered state rather than an immediate layer displacement driven by the shear phonon. Together with a photodriven effective hole-doping of the valence band and a destruction of the local order of the polarization, such modulation can facilitate a shear displacement to the positions of the metastable phase. The shear displacement associated with the formation of the metastable phase saturates at approximately 8 pm; this shear is smaller than the expected 37 pm for the 1T* phase.

## Methods

### Experimental Method

Single crystals of *Td*-WTe_2_ (2D Semiconductor, USA) were carefully sliced along the layers into TEM samples with a diamond knife mounted on a ultramicrotome (Leica). The TEM samples were placed on single layer graphene TEM grids (Ted Pella). All samples were characterized by energy loss spectroscopy (EELS), and the thickness was estimated using a simulated 94 nm inelastic mean free path (IMFP) for 200 keV electrons in WTe_2_.^38^ The time-resolved diffraction experiment was performed in an ultrafast electron microscope operating at 200 kV using a hybrid pixel detector (CheeTah1800, Amsterdam Scientific Instruments). The experimental setup is described in detail in ref (39). The sample was excited by a λ = 515 nm 300 fs laser (Tangerine, Amplitude Systemes) focused to a spot with fwhm of ∼120 μm. WTe_2_ samples were excited at a repetition rate of no more than 70 kHz to allow for complete relaxation in-between shots. The electron bunches were generated through photoemission from a guard ring LaB_6_ cathode by a 258 nm laser pulse coupled to the pump pulse. The temporal width of the electron bunches used in the diffraction experiments was approximately 1.4 ps, as characterized by photoinduced near field electron microscopy.^40^ A JEOL liquid nitrogen cooling holder (EM-21090) was used to characterize the sample at 110 K. When not otherwise indicated, the experiments were performed at room temperature using a double tilt sample holder (JEOL).

### Computational Method

The band-structure and density of states presented in Figure 6 and the structural dependence on hole-doping presented in Figure 7 were calculated using the VASP implementation of DFT.^41,42^ Exchange and correlation was treated with the GGA functional by Perdew, Burke, and Ernzerhof^43^ and with Grimme’s D3 treatment of dispersion interaction,^44^ including Becke-Johnson damping.^45^ We used an energy cutoff for the plane-wave expansion of 446 eV. The Brillouin-zone was sampled with 12 × 6 × 3 k-points in a Γ-centered mesh. Spin–orbit coupling is included. Structural relaxations were stopped when the difference in total free energy between two relaxation steps was less than 1e^–6^ eV.

We performed nonadiabatic molecular dynamics using time-dependent density-functional theory in conjunction with Ehrenfest dynamics as implemented in the TDAP package,^46^ built on top of the Siesta electronic structure code.^47^ The simulations were performed using a supercell of 4 × 2 × 1 unit-cells of *Td*-WTe_2_, comprising 96 atoms, sampling only the Γ-point of the supercell. The exchange-correlation energy was described within the adiabatic GGA approximation, according to Perdew, Burke, and Ernzerhof.^43^ As basis set a double-ζ + polarization orbital representation was used. The mesh cutoff for grid-integration was set to 129 Ry. Time-propagation was performed with a 6.045 atto-second time-step, the ionic motion is propagated within the Ehrenfest molecular dynamics scheme using the Verlet algorithm.^48^ The electromagnetic field of the pump-pulse is represented as a vector potential in the velocity gauge. The external field, represented in the velocity gauge, enters the calculation as a vector potential *A*(*t*) = −∫_*t*_0__^*t*^sin(*ωt*′) *dt*′, where  = 0.015 V/Å, and the angular frequency corresponds to ℏω = 2.4 eV. Note that this represents a rectangular pulse shape, in our simulations represented as a continuous pulse starting from *t* = *t*_0_ and ending at *t* = *t*_end_; that is, no Gaussian envelope is applied. See the Supporting Information for details on power density and intensity in relation to a Gaussian envelope. We calculate the time-evolution of the 16 individual Δ_1_ and Δ_2_, as defined by the *b* axis displacement of Te atoms in adjacent layers. Average quantities are calculated according to ⟨Δ_1_(*t*)⟩ = 1/*N*∑Δ_1_^*i*^(*t*) and ⟨Δ_2_(*t*)⟩ = 1/*N*∑Δ_2_^*i*^(*t*) (see Figure 1 for definition) represented in the supercell.

## Supporting Information Available

Temporal evolution of diffraction intensity at the [025] zone axis, normalized modulation of the structure factor by a shear phonon, intensity trace of the 130 diffraction spot in the temporal range up to 200 ps, shear phonon amplitude as a function of pump fluence, TEM images of samples, change in normalized intensity for several diffraction spots as a function of shear displacement of the middle layer toward the negative *b* direction, and illustration of diffuse scattering and the fitting methods (PDF)
