# Supplementary material for: Manipulation of Stacking Order in Td-WTe2 by Ultrafast Optical Excitation
Source: ACS Nano. 2021 Apr 29;15(5):8826–35. doi: 10.1021/acsnano.1c01301 (PMC8291768; doi:10.1021/acsnano.1c01301)
Supplement: Supplementary file 1 — nn1c01301_si_001.pdf [file nn1c01301_si_001.pdf]

Supplementary materials to:

Manipulation of Stacking Order in *Td*-WTe_2 by Ultrafast Optical Excitation

Shaozheng Ji[1], Oscar Grånäs[2], and Jonas Weissenrieder[1*]

[1]Materials and Nano Physics, School of Engineering Sciences, KTH Royal Institute of Technology, SE-100 44 Stockholm, Sweden

[2]Division for Materials Theory, Department of Physics and Astronomy, Uppsala University, SE-751 20 Uppsala, Sweden

Phonon excitation at low temperature

The experiment was performed at 110 K using a liquid nitrogen cooling holder. A 41 nm thick sample was pumped with 1.9 mJ/cm^2 laser fluence at 515 nm. The sample was tilted to the [025] zone axis for selected area electron diffraction. The diffraction pattern is shown in Figure S1b with the corresponding simulated diffraction pattern (by SingleCrystal) overlayed. The experimental and simulated patterns show good agreement. Several indexed diffraction spots are shown in Figure S1b. The intensity evolution of two diffraction spots, following photo-excitation, is shown in Figure S1a. The temporal traces show oscillations with opposite phase. The extracted oscillation frequency from the fast Fourier transform shown in Figure S1c is 0.27 THz. A slightly higher frequency than the 0.23 THz obtained in the room temperature experiments shown in Figure 2 of the main text. The phonon is hardened at low temperatures. The initial phase of the oscillation at 110 K is best described by a cosine function, similar to what is observed at room temperature, and is in agreement with the displacive excitation of coherent phonons (DECP) mechanism.

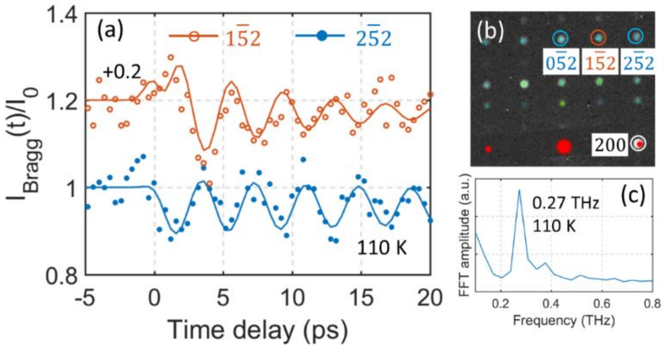

Figure S1. (a) Temporal evolution of the diffraction intensity of spots $1\bar{5}2$ and $2\bar{5}2$. The sample was kept at 110 K and pumped with a 1.9 mJ/cm^2 laser fluence. (b) overlayed images of the experimental and the simulated diffraction patterns at the [025] zone axis. (c) Fast Fourier transform (FFT) of the results in (a). The obtained frequency is 0.27 THz.

Structure model of shear mode and structure factor calculation

The excited optical phonon which is called 'shear' mode here can be described as an antiphase motion of adjacent layers of WTe_2 along the **b** axis. The initial shear direction is depicted by the arrows in Figure S2b. For simplicity, we can assume that the middle layer shear towards the negative **b** direction relative to the top and bottom layer. Since the phonon can be explained by the DECP model, the initial stacking position for the middle layer is at the rightmost position of phonon. The metastable

equilibrium position of the shear phonon exhibits a negative **b** offset for the middle layer of unit cell. Based on this, the shear phonon can be described by the following equation:

$$d = \mathrm{A}\cos\left(\frac{2\pi t}{T}\right) - \mathrm{A} \qquad \textbf{(eq. 1)}$$

where *A* is the amplitude of phonon, *T* is the period, and *d* is the displacement.

By assuming T = 4 ps and A = 2.5 pm we can calculate the modulation of the structure factor by the shear phonon according to the following equation:

$$F_{hkl}(\boldsymbol{r}) = \sum_j f_j \exp\left[-2\pi i(hkl)\cdot \boldsymbol{r_j}\right] \qquad \textbf{(eq. 2)}$$

Where f_j is the scattering factor and $\boldsymbol{r_j}$ is the position of the *j*th atom.

The intensity of the (hkl) Bragg diffraction spot, I_{Bragg}, is proportional to the square modulus of the structure factor F_{hkl}. The atomic position for ground state Td-WTe_2 is obtained from Ref. [1]. When the shear phonon is excited, the atomic position of middle layer is modulated by *d* from **eq. 1**. The relative intensity modulation by the shear phonon were calculated for six diffraction spots and the results are shown in Figure S2a. The simulated results show that the expected oscillations in diffraction intensity from the shear phonon is in agreement with the experimental results shown in Figure 2 and in Figure S1. Note that the Debye-Waller effect is not considered in the simulation.

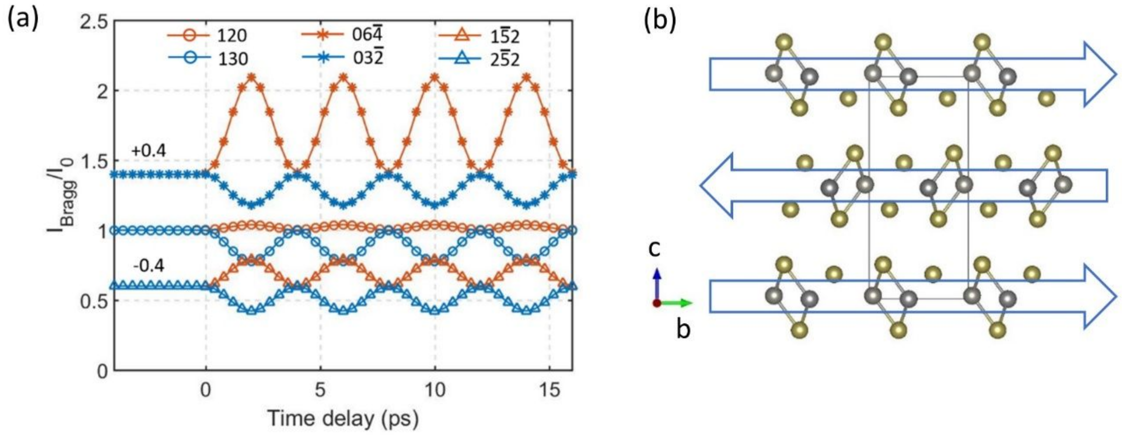

Figure S2. (a) Normalized modulation of the structure factor for several diffraction spots by a shear phonon along the **b** axis. The initial shear direction of the antiphase oscillation of the adjacent layers in WTe_2 is illustrated by the arrows in (b). The period of the phonon in the model is assumed to be 4 ps and the amplitude of the shear phonon displacement 2.5 pm.

Lifetime of photo-induced metastable phase

The photo driven metastable phase persists for at least 200 ps as shown by the intensity of the 130 Bragg diffraction spot in Figure S3.

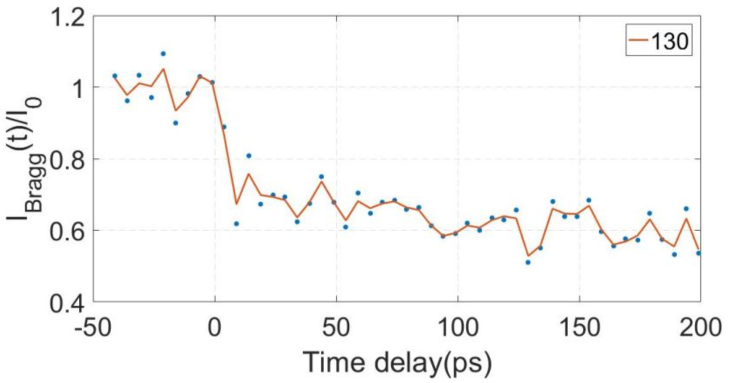

Figure S3. Intensity trace of the 130 diffraction spot in the temporal range up to 200 ps at 2.5 mJ/cm^2 pump fluence.

Phonon amplitude as a function of pump fluence

The phonon amplitude is characterized by the amplitude of the intensity oscillation of the **130** Bragg spot in a sample with < 10 nm thickness. To reduce the influence of photo driven phase transition, the amplitude is extracted from the time range 6 - 10 ps after time zero. The amplitude of the intensity oscillations as a function of pump fluence is shown in Figure S4. Below 0.5 mJ/cm^2 we observe a near linear increase in amplitude with increasing pump fluence. No significant increase in amplitude is observed after 0.5 mJ/cm^2, indicating a complex behavior which may be influenced by the formation of a metastable phase.

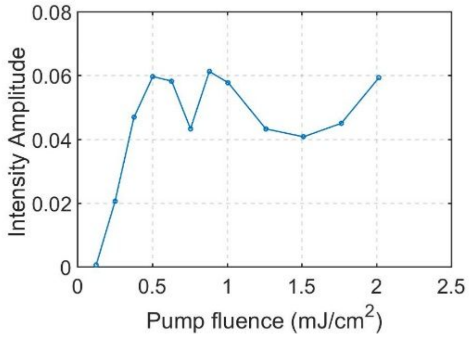

Figure S4. Intensity oscillation amplitude obtained from the 130 Bragg diffraction spot as a function of pump fluence in a thin sample (below 10 nm in thickness). The amplitude is extracted from the time range 6 - 10 ps after time zero.

Image of samples with and without strong shear mode excitation

The presence of defects is arguably the reason behind the suppression of the shear phonon presented in Figure 4 of main text. TEM images of a sample with and without strong shear phonon excitation are shown in Figure S5. The flat sample in Figure S5a shows strong photo-excited phonon signal. While the sample in Figure S5b exhibit defect and small ripple domains.

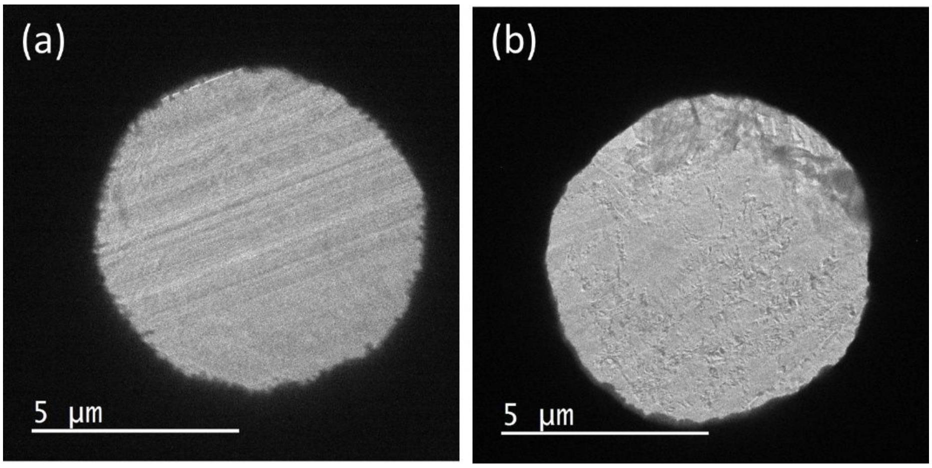

Figure S5. TEM images of two samples. In sample (a), the photoexcited shear phonon is strong. While in sample (b), the shear phonon is strongly suppressed. The presence of defects in (b) is arguably the reason behind the suppression of the shear phonon.

Calculation of shear displacement in the metastable phase

The formation of the metastable phase can be modeled by shearing the middle layer of the unit cell towards the negative **b** direction. The lattice arrives at the metastable equilibrium stacking position through a process with a time constant of ~5 ps. The shear displacement increases linearly with pump fluence until saturation at a pump fluence in excess of ~2.3 mJ/cm^2 (as shown in Figure 5 of the main text). In order to extract the saturation shear displacement, the normalized change in intensity as a function of shear displacement of the middle layer in the *Td*-WTe_2 unit cell along the negative **b** direction is calculated for several diffraction spots (solid lines in Figure S6). The intensity of the 130, 150, and 170 spots decrease with the increase of shear displacement while the intensity of the 120 spot increase.

From the intensity gap between 3 ps and 30 ps as shown in Figure 5a of main text the corresponding shear displacement of the metastable phase can be obtained from the solid curves in Figure S6. For conditions with saturated shear displacement, we extract normalized intensities for the 120, 130, 150, and 170 Bragg diffraction spots (indicated by square symbols with error bars in Figure S6). We extract an average saturation shear displacement of ~8 pm by considering the four diffraction spots.

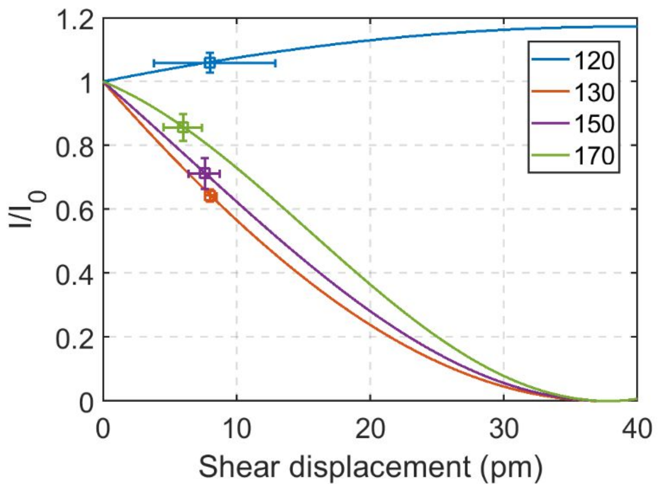

Figure S6. Simulated change in normalized intensity for several diffraction spots as a function of shear displacement of the middle layer in the *Td*-WTe_2 unit cell along the negative **b** direction. The square symbols with error bars are extracted from the maximized intensity gap observed between the 3 ps and 30 ps traces shown in Figure 5a. Influence of the Debye-Waller effect was considered for the intensity decay at 3 ps.

Diffuse scattering

The intensity of the diffuse scattering between Bragg diffraction spots was traced following photo-excitation. The results are summarized in Figure S7. As can be seen in Figure S7c, the diffuse intensity in Regin I and II increase within 2 ps after photo-excitation which is similar to the instrument response time.

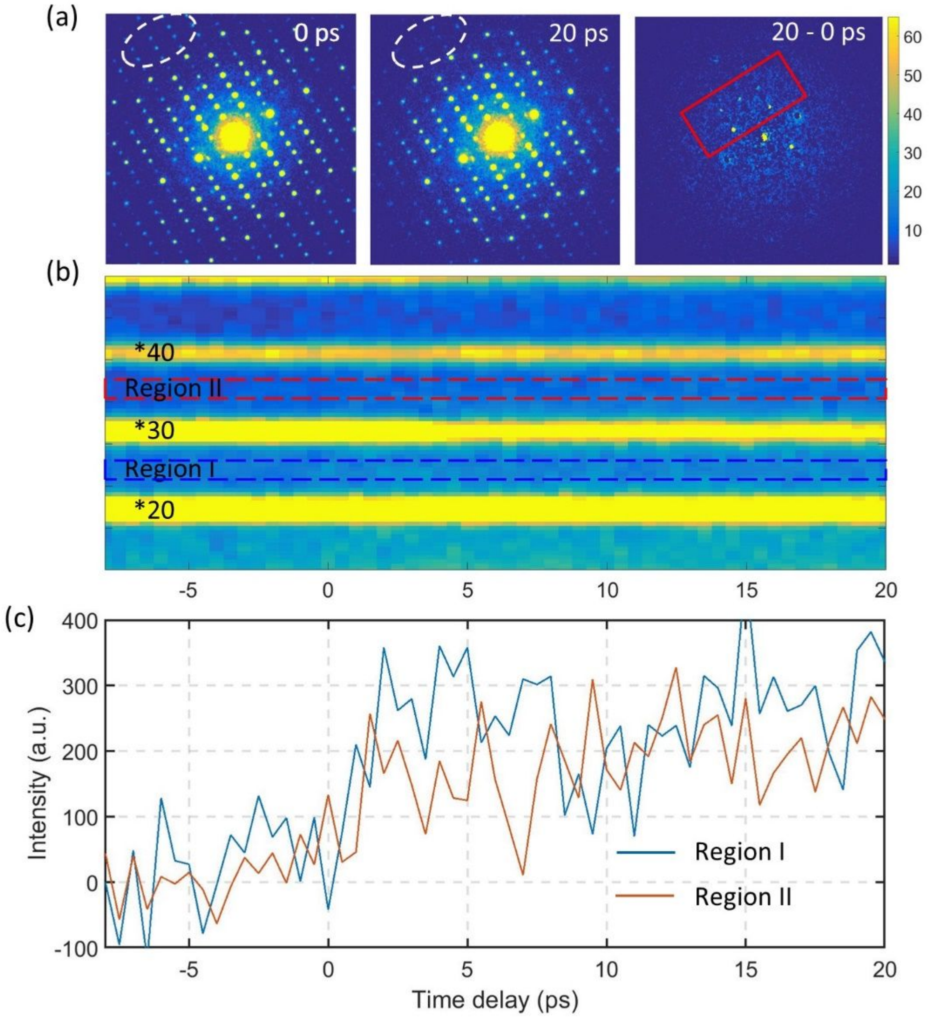

Figure S7. (a) Diffraction patterns obtained at 0 ps and 20 ps after photo excitation with 1.5 mJ/cm^2 laser fluence. The dashed ovals indicate regions with dramatic decrease in diffraction intensity at 20 ps. In the subtracted diffraction pattern (20 ps - 0 ps) we observe diffuse scattering at a wide range of momentum positions. The three diffraction patterns in (a) use the same color intensity scale. The red rectangle indicates the selected area used to trace the diffuse scattering intensity by projection along the **a** axis. This area includes the *20, *30 and *40 diffraction spots. The resulting projection is shown in (b). (c) shows the diffuse scattering intensity evolution in regions I and II indicated in (b). The average intensity before time zero is subtracted. Diffuse scattering appears quickly after the photo-excitation.

Method for data fitting

1. Fitting method used in Figure 2a and c.

The temporal trace of the diffraction intensity is fitted using a least squares method by a combination of an exponential decay with a sub-ps time constant and the 0.23 THz shear phonon. The thermal effect is considered by an exponential decay with sub-ps time constant as extracted from the change in intensity of the 400 diffraction spot shown in Figure 3b of main text. As presented in the '*Structure model of shear mode and structure factor calculation'* section of the text, the intensity oscillation due to the shear phonon can be described by a cosine function. The functions used in the fitting procedure are listed below.

Function for fitting the evolution of the 120, $06\bar{4}$, $1\bar{5}2$ diffraction intensity:

$$z(t) = \left[1 + A + A * \exp\left(-\frac{t}{\tau_1}\right)\cos\left(\frac{2\pi t}{T} + \pi\right)\right] * [1 - B + Bexp\left(-\frac{t}{\tau_2}\right)] \quad \textbf{(eq. 3)}$$

Function for fitting the evolution of the 130, $03\bar{2}$ $2\bar{5}2$ diffraction intensity:

$$z(t) = \left[1 - A + A * \exp\left(-\frac{t}{\tau_1}\right)\cos\left(\frac{2\pi t}{T}\right)\right] * [1 - B + Bexp\left(-\frac{t}{\tau_2}\right)] \quad \textbf{(eq. 4)}$$

Where A is the amplitude of phonon, T is the period of the shear phonon, τ_1 is the damping constant of the shear phonon, B is the intensity decay due to the Debye-Waller effect, τ_2 is the decay time constant of Debye-Waller effect, and t is time. τ_2 was set to be 0.5 ps as obtained from fitting of 400 diffraction spot intensity evolution.

Table S1. Fitting parameters for Figures 2a and c of the main text.

Spots	A	B	τ_1 (ps)	T (ps)
**120 spot**	0.05	0.01	50	4.1
**130 spot**	0.07	0	40	4.1
**06-4 spot**	0.31	0.19	32	4.2
**03-2 spot**	0.10	0	50	4.2

2. Fitting method used in Figure 3a

The fitting of 130 and 140 diffraction intensity of the 8 nm thickness sample use two exponential functions and a shear phonon, a fast exponential decay with a sub-ps time constant and a slower decay/rise time constant. Since the formation of metastable phase impacts the phonon amplitude and frequency, the first and second period of phonon was assumed to have a different frequency and amplitude. After the initial two periods, the phonon was assumed to have a damped amplitude with a certain frequency. The exact fitting functions are listed below.

Functions for fitting the evolution of the 140 diffraction intensity:

$$z(t) = \left[1 + C - Cexp\left(-\frac{t}{\tau_3}\right) + A_0 + A_0\cos\left(\frac{2\pi t}{T_1} + \pi\right)\right] * [1 - B + Bexp\left(-\frac{t}{\tau_2}\right)],\ t < T_1 \quad \textbf{(eq. 5)}$$

$$z(t) = \left[1 + C - Cexp\left(-\frac{t}{\tau_3}\right) + A_0 + A\cos\left(\frac{2\pi(t - T_1)}{T_2} + \pi\right)\right] * [1 - B + Bexp\left(-\frac{t}{\tau_2}\right)],$$

$T_1 \leq t < T_1+T_2$ **(eq. 6)**

$$z(t) = \left[1 + C - Cexp\left(-\frac{t}{\tau_3}\right) + A_0 + A\exp\left(-\frac{t - T_1 - T_2}{\tau_1}\right)\cos\left(\frac{2\pi(t - T_1 - T_2)}{T_3} + \pi\right)\right] * [1 - B + Bexp\left(-\frac{t}{\tau_2}\right)],$$ $t \geq T_1+T_2$ **(eq. 7)**

Functions for fitting the evolution of the 130 diffraction intensity:

$$z(t) = \left[1 - C + Cexp\left(-\frac{t}{\tau_3}\right) - A_0 + A_0\cos\left(\frac{2\pi t}{T_1}\right)\right] * [1 - B + Bexp\left(-\frac{t}{\tau_2}\right)],$$ $t < T_1$ **(eq. 8)**

$$z(t) = \left[1 - C + Cexp\left(-\frac{t}{\tau_3}\right) - A_0 + A\cos\left(\frac{2\pi(t - T_1)}{T_2}\right)\right] * [1 - B + Bexp\left(-\frac{t}{\tau_2}\right)],$$ $T_1 \leq t < T_1 + T_2$

(eq. 9)

$$z(t) = \left[1 - C + Cexp\left(-\frac{t}{\tau_3}\right) - A_0 + A\exp\left(-\frac{t - T_1 - T_2}{\tau_1}\right)\cos\left(\frac{2\pi(t - T_1 - T_2)}{T_3}\right)\right] * [1 - B + Bexp\left(-\frac{t}{\tau_2}\right)],$$

$t \geq T_1 + T_2$ **(eq. 10)**

Where A_0 is the amplitude of the shear phonon in the first oscillation, A is the amplitude of the shear phonon after the first period, B is the intensity decay due to the Debye-Waller effect, C is the intensity decay due to the formation of the metastable phase. T_1 is the first period of the shear phonon, T_2 is the second period of the shear phonon, T_3 is the period after the first two periods. τ_1 is the damping constant of the shear phonon, τ_2 is the decay time constant due to the Debye-Waller effect. τ_2 was set to be 0.5 ps as obtained from the fitting of the 400 diffraction spots intensity evolution. τ_3 is the time constant for the formation of the metastable phase. t is time.

Table S2. Fitting parameters for the 8 nm thick sample in Figure 3a of the main text.

**Spots**	**A**	**B**	**C**	**A_0**	**τ_1(ps)**	**τ_3(ps)**	**T_1 (ps)**	**T_2 (ps)**	**T_3 (ps)**
**130 spot**	0.34	0.05	0.33	0.05	9.4	6.0	4	4.6	5.5
**140 spot**	0.20	0.08	0.10	0.05	17.3	4.2	4	4.6	5.5

Simulated pulse shape *vs* experimental conditions

The experimental pulse profile is approximately described by a Gaussian function. The onset of the Gaussian pulse is slow, rendering the simulations exceedingly heavy in order to cover the time when the Gaussian profile has insignificant power density. In order to make simulations of the initial pump dynamics feasible, we have employed a rectangular pulse shape starting at $t = t_0$ ending at $t = t_1$, in our simulations this is equivalent to continuous light conditions between t_0 and t_1. It is clear from the intensity profile in figure S8, panel a), that this representation captures approximately the rate of energy increase at the surface of the sample. Using the same peak power density as the Gaussian beam profile at 2.0 mJ/cm^2, for a rectangular pulse shape, we reach 1.3 mJ/cm^2 after 200 fs, and 1.6 mJ/cm^2 after 250 fs, which is the maximum time reported in the main text. Our simulations use periodic boundary conditions; hence the field is the same in the entire crystal. Considering the slight attenuation of the field in the experimental setup, these conditions are representative of experimental situation.

Table S3. Envelopes are described as G for Gaussian, with standard deviation σ = 127 fs = FWHM/2.355, for a FHWM of 300 fs. R indicates a rectangular pulse shape, described by a boxcar function, $A(H(t-t0)-H(t-t1))$, in our simulations equivalent to a CW-illumination with amplitude A, between t_0 and t_1.

Envelope (type, fs)	G(σ=127)	G(σ=127)	G(σ=127)	G(σ=127)	R(t=200)	R(t=250)	R(t=300)
Total Intensity (mJ/cm^2)	0.5	1.0	2.0	4.0	1.3	1.6	2.3
Peak Power Density (GWcm^2)	1.57	3.4	6.27	12.53	6.27	6.27	6.27

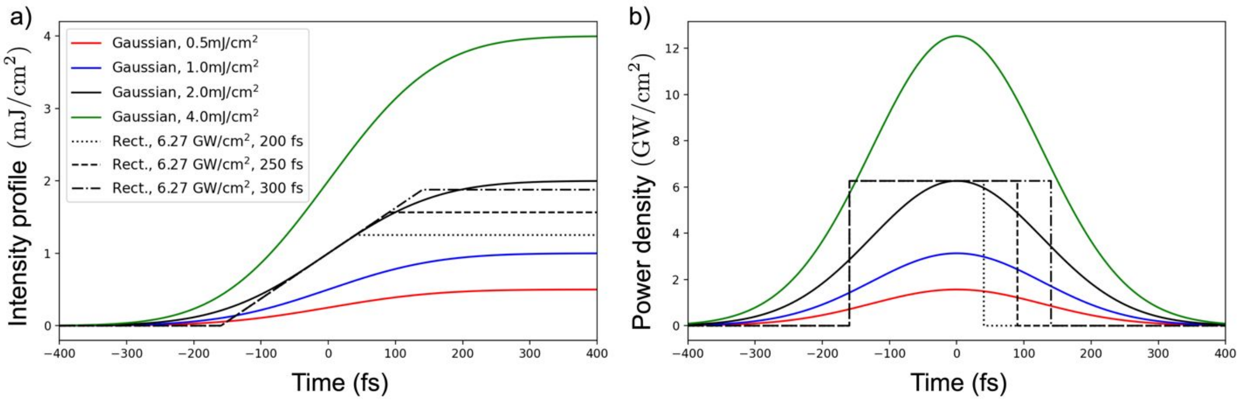

Figure S8: Panel a) shows the intensity on the sample for different characteristics of the pump. Panel b) shows the power density profile. To facilitate comparison to the Gaussian profiles, the rectangular pulse is shifted so that intensity equals to 1 mJ at t = 0.

References

1. Mar A, Jobic S, Ibers JA. Metal-Metal *vs* Tellurium-Tellurium Bonding in WTe_2 and Its Ternary Variants $TaIrTe_4$ and $NbIrTe_4$. *Journal of the American Chemical Society*, **1992**, 114(23)**:** 8963-8971.
